# Low Dose and Non-Targeted Radiation Effects in Environmental Protection and Medicine—A New Model Focusing on Electromagnetic Signaling

**DOI:** 10.3390/ijms231911118

**Published:** 2022-09-21

**Authors:** Carmel Mothersill, Alan Cocchetto, Colin Seymour

**Affiliations:** 1 Department of Biology, McMaster University, Hamilton, ON L8S 4K1, Canada; 2 National CFIDS Foundation, 285 Beach Ave., Hull, MA 02045-1602, USA

**Keywords:** ionizing radiation, UVA, acoustic signals, non-targeted effects, variable response model, bystander signals

## Abstract

The role of signalling in initiating and perpetuating effects triggered by deposition of ionising radiation energy in parts of a system is very clear. Less clear are the very early steps involved in converting energy to chemical and biological effects in non-targeted parts of the system. The paper aims to present a new model, which could aid our understanding of the role of low dose effects in determining ultimate disease outcomes. We propose a key role for electromagnetic signals resulting from physico-chemical processes such as excitation decay, and acoustic waves. These lead to the initiation of damage response pathways such as elevation of reactive oxygen species and membrane associated changes in key ion channels. Critically, these signalling pathways allow coordination of responses across system levels. For example, depending on how these perturbations are transduced, adverse or beneficial outcomes may predominate. We suggest that by appreciating the importance of signalling and communication between multiple levels of organisation, a unified theory could emerge. This would allow the development of models incorporating time, space and system level to position data in appropriate areas of a multidimensional domain. We propose the use of the term “infosome” to capture the nature of radiation-induced communication systems which include physical as well as chemical signals. We have named our model “the variable response model” or “VRM” which allows for multiple outcomes following exposure to low doses or to signals from low dose irradiated cells, tissues or organisms. We suggest that the use of both dose and infosome in radiation protection might open up new conceptual avenues that could allow intrinsic uncertainty to be embraced within a holistic protection framework.

## 1. Introduction to Low Dose and Non-Targeted Radiobiology

### 1.1. General Background

The mechanisms underlying low dose and low dose rate effects of ionising radiation are not fully understood but are now accepted to be quite different to high dose effects. Low dose is generally defined as <100 mGy while low dose rate is <6 mGy/h [1]. Signalling mechanisms induced by low doses determine responses of cells tissues and organisms [2,3,4]. Non-targeted effects (NTE), which include the bystander effect and delayed non-clonal expression of reproductive death, mutation and chromosomal aberrations dominate the response after low doses [4]. Although NTE are seen after high doses they contribute proportionally less to the total response. The evidence for both good and bad low dose effects is presented in a comprehensive review of the animal data by Tang et al. [5] who concluded “all the radiation exposures induced either bionegative or biopositive effects on fertility, tumorigenesis, and lifespan depending on genetic background, age, sex, nature of radiation exposure (i.e., acute or chronic irradiation), type of ionizing radiation applied, experimental design and statistical methodology used”. Until there is a dominant or unified message, the responses are different in different cells, tissues, organs or organisms. A major question we intend to address in this paper is how the different responses to radiation get coordinated and whether if these coordinating factors were better understood, we could impact the treatment of diseases including cancer and CFS, where there is a public perception but quite controversial and often contradictory evidence that LDR is implicated in the aetiology.

### 1.2. The Importance of Signalling

A cornerstone of the thinking being discussed in this paper is that signalling coordinates higher level response. We suggest that the effect of low dose radiation is due to the dominant signal or message produced (“dominant” not “only”). Competing messages are “noise”. The consensus in the field is that signalling pathways induced by stressors including radiation are the key to understanding both the system level coordination of low dose responses and the factors determining outcomes. These pathways constitute the messaging and how they play out will be a consequence not just of dose, but of time, context, and history. In other words the response may depend on the experiences and memories of the recipient of the dose, be it a cell or an ecosystem. Many of these signalling pathways are very well documented, e.g., [2,3,4], but the idea of them competing is less thought about [6,7]. Recent developments in pathway and network analysis in system biology [8,9,10,11] as well as the approaches favoured in mathematics and economics such as complexity and chaos modelling (forecasting) offer hope that holistic approaches may become more widely used in future [12,13,14].

Signalling pathways thus provide a means for coordinating responses but identification of a signaling pathway does not inform about the ultimate outcome other than in a probabilistic or stochastic sense [15,16,17,18,19]. A greater understanding of the steps involved in the signaling process might make outcomes more predictable. The issue of uncertainty is discussed in Mothersill and Seymour 2022 [20]. These ideas are fiercely unpopular among those who wish to promote a threshold model of radiation protection because any possibility that radiation might cause any adverse low dose effects, means that a precautionary approach is likely to be retained when setting dose limits [21,22,23,24]. The reality is that low dose hypersensitivity exists [25,26,27,28] and non-targeted effects (discussed in the next section), can result in both “good” and “bad” effects [2,29,30,31,32]. The debate should not really be centered on whether low doses of radiation are good or bad but should be concerned with how systems deal with low doses of stressors and whether improved modelling or approaches such as adverse outcome pathway (AOP) analysis can improve our ability to understand and thus predict individual responses. Moves to do this are happening now [33,34,35]. It is also worth noting here that in environmental radiation protection where survival of the individual (or prevention of every single cancer) is not the protection goal, threshold models are widely used. These models predict doses limits which protect populations or biodiversity rather than individuals.

### 1.3. Non-Targeted Effects

Among the most important low dose signalling and communication mechanisms are the non targeted effects (NTE) which predominate in the low dose range and refer to events occurring in cells, tissues or organisms which did not receive a dose of radiation but received signals from irradiated cells tissues or organisms (see [36,37,38] for recent reviews). NTE used to be thought of as “bad” effects which somehow increased the “size of the target” (bystander type effects) or the mutation tolerance of the system (genomic instability type effects). Now however we have a much more nuanced holistic view and regard NTE as a response to an insult or stress in the system rather than an insult in themselves. Depending on the level of organisation examined, responses can appear to be good or bad but it is very fluid and grey rather than black and white. The implications for risk attribution and risk analysis of the multiple outcome options available to systems exposed to low doses of stressors including radiation, are discussed in a recent publication by the authors [2].

### 1.4. Candidate Signals-Chemical and Physical

The signals, and downstream events in cells affected by bystander signals have been discussed in many reviews (e.g., [38,39,40]). Signals include calcium, reactive oxygen species (ROS), nitric oxide (NO), and more recently identified physical signals including UVA and sound. Exosomes and other extracellular vesicles appear to be important conveyers of signals from targeted to bystander cells and in recipient cells, membrane channels, TGFb and p53 are among agents involved in transduction of bystander, with mitochondrial metabolism known to be a key factor in determining outcomes.

Our focus in this paper is on the physical signals and possible transduction pathways of these signals. The demonstration that photon emission from irradiated cells can induce bystander effects in non-exposed cells is new but there is a considerable amount of evidence in the literature for photon emission from both living and dead organic material and in living organisms there is evidence that the photon emission can be linked to signaling processes.

### 1.5. History of Physical Signal Discoveries

Since the earliest reports in the 1920s, there have been many papers documenting low intensity photon emission from plant, animal, and human-derived material. This history and more recent evidence are reviewed in Gurwitsch [41]. Popp et al., 1984 [42], Gurwitsch 1920 [43], Baron 1926 [44], Bajpai et al., 1991 [45] found evidence in plants. Animal emissions were recorded by Devaraj et al., 1991 [46], Evelson et al., 1997 [47], and Van Wijk et al., 2014 [48], and emissions from human or murine derived material were detected by Niggli 1996 [49], Niggli et al., 2008 [50], and Van Wijk et al., 2013 [51]. These are often referred to as ultraweak luminescence or biophoton emission however in this paper we refer to them as photon emissions from biological material. Photon emission at very low fluxes has also been observed in the absence of a stimulus. This is referred to as spontaneous photon emission [52]. These weak electromagnetic fields are thought to be informational signals intended to facilitate inter- and intra-cellular communication within a population of cells, tissues or organisms [41,53,54,55]. Photon emission can also be elicited in response to a stimulus. The stimulated emission is generally much bigger. Previous work done in our laboratory [56,57] showed that beta irradiation of cells (human keratinocytes) led to significant photon emission in the blue and ultraviolet A (UVA) range. Photon emission from biological materials has also been shown to be induced by chemical agents and is therefore likely to be a generic stress response to foreign stimuli [58]. Van Wijk and colleagues have even used it as a marker of disease and stress states in animals and humans although this type of application is highly controversial [59].

### 1.6. Physical Signals Can Trigger the Bystander Effect

As is the case with spontaneously emitted photons, previous work from our lab has shown that the radiation-induced UV photons can act as bystander signals activating radiation–like responses in non-targeted cells [60]. The experiments involved placing a layer of tritium-incubated cells, under another layer of untreated cells separated by a barrier such that the upper cells were only exposed to the subsequent radiation-induced UV, not the initial beta radiation from the tritium. A bystander effect was observed in the upper layer of non-beta exposed cells proving that UV induced cell communication occurred. When a UV filter was inserted between the layers, the bystander effect, i.e., the induced cell communication, was not observed. We previously observed UV photon emission from a number of organic materials including plastics and dried tissues [61], so one mechanism of UV emission is probably simply a consequence of electron rearrangement, e.g., excitation decay, after ionization events in atoms and/or molecular structures. Recent experiments confirmed that gamma ray exposure of cells also leads to photon emission [62]. Attempts to demonstrate photon emission using an alpha emitter (radium) suggest that radium does not induce photon emission (Hossian paper in prep). The demonstration that electromagnetic signals resulting from physicochemical events in atoms and molecules experiencing ionizing radiation energy deposition is interesting.

### 1.7. Acoustic Signals

The other physical signal which we have considered is an acoustic signal [63]. Radiation induced sonic emissions are in widespread use for non-invasive structural testing. Our laboratory first became interested in the possibility of acoustic signalling as a form of ionising radiation-induced bystander communication when studying the mechanism of fish to fish bystander signalling. A test involving putting irradiated fish in a small aquarium inside a larger aquarium containing non-irradiated fish demonstrated that the signal could pass through glass (discussed in [20]). This provided the first evidence that a water-bourn chemical did not mediate the transmission of the signal in these experiments. Further experiments showed that even when the inner aquarium was covered in aluminium foil, the signal was still transmitted leaving us with the possibility that an acoustic signal was involved. As it was not then possible to test this possibility measurements were done to test whether a hypothetical acoustic signal could travel from the irradiated fish to the non-exposed fish. This possibility was not excluded by our measurements. Subsequently, in collaboration with acoustic physicists at the University of Cambridge, acoustic emissions have been measured from irradiated cells which are different to those coming from unirradiated cells and while these acoustic signals have not been proven by themselves to induce a bystander effect, the magnitude of the signal is associated with the magnitude of the bystander effect in two different cell lines (Matarèse et al., revision submitted to IJRB).

Acoustic emissions may have pleiotropic effects on affected cells through several mechanisms, one of which is the generation of reactive oxygen species which accompanies thermoelastic expansion. ROS is a well-known bystander signal [64,65,66]. The generation of ROS by thermoelastic expansion is well characterised and there is evidence for free radical production in aqueous and nonaqueous solutions exposed to ultrasound [67,68,69]. Recent experiments link the generation of intracellular ROS by low intensity ultrasound to cell killing in a number of cell lines [70]. It is possible that both the light and acoustic emissions may be related to the Cerenkov effect since this is physically and mathematically related to both acoustic shock waves and blue light emissions. However, further discussion of this is outside the scope of this paper.

The next question is how these events translate to biological effects in both the exposed cells, tissues and organisms and their non-targeted bystanders.

## 2. Downstream Processing of Electromagnetic Signals

**Figure 1:**
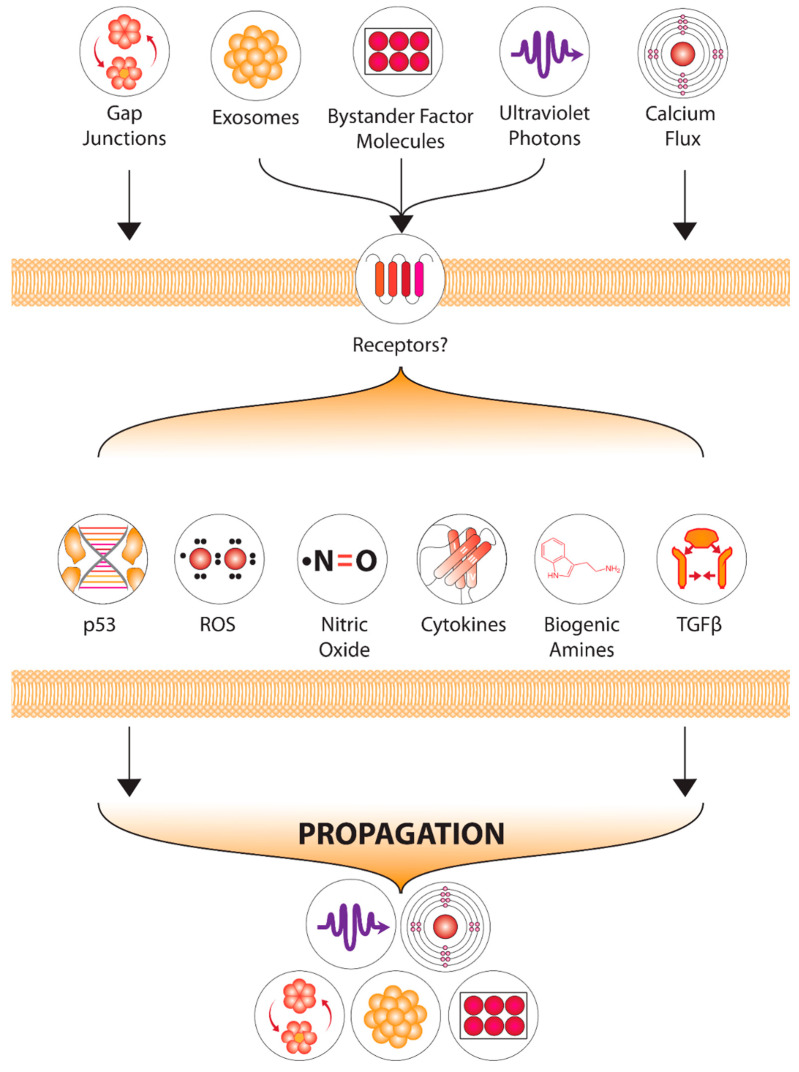
Our current understanding of the multiple factors involved in the radiation-induced bystander communication process.

While we know many steps in the downstream processing of bystander signals, as shown in Figure 1, there are two overall issues that we consider of over-riding importance. One is that after low doses and in non-targeted cells, the response to the signal, in addition to the features of the signal itself, appears to determine outcomes. The second issue is that the outcome has to be viewed in context—elimination or retention of affected individuals may be beneficial or may adversely affect the system in different ways at different times. This means a different approach may be needed if trying to determine “risk”—holistic rather than reductionist with the introduction of new models that factor in multiple outcomes and uncertainty modeling. As suggested earlier, the idea that outcomes following exposure to stressors depend on the response rather than the dose alone, are deeply worrying to those trying to set dose limits. It is easy to measure dose and easy to apply dose limits based on either some form of threshold model or a linear-no-threshold model with built in safety factors. It is much harder to deal with messy biology where multiple factors can modulate risk or outcome for better or worse. Epidemiology is a very blunt tool in the low dose range of exposure leaving the public very concerned about the size of the uncertainties. This is discussed later in the section on radiation protection but generally it is assumed that the worst-case scenario is the only true scenario. Some clues to possible approaches may be gained from a consideration of the field of chemical ecology or from microbiome research.

## 3. Chemical Ecology, Mychorrysal and Microbiome Evidence

These are large areas of research which are becoming very popular. Chemical ecology and related studies of intra- and inter-organismal signalling mediated by fungi and bacteria such as microbiome research and mycelium communication systems deal with chemical and physical signals and receptors at the level of cells or organisms. The signals are referred to as “infochemicals” and studies suggest that far from stressors leading to uncertain outcomes, the responses are highly controlled [71,72,73,74,75]. Chemicals can be identified and tested, clear hypotheses can be formulated. All that is required is a shift in thinking so that the response of the system to a basket of stressors dominates the debate rather than the current silo approach of looking at the dose of single stressors in isolation. In chemical ecology, the chemicals function as highly sensitive communicators of information to receptors [76,77,78,79,80,81]. These may be predators responding to messages alerting them to the presence of prey (known as kairomones), prey responding to signals from predators (allomones) or signals from potential mates (pheromones). Chemicals released by plants warn other plants to produce distasteful chemicals to deter grazing animals in a similar way [82,83,84,85]. Recently there have been multiple accounts in both peer reviewed journals and in the popular press about the so-called “wood-wide web” [86,87,88], detailing the complex way trees communicate using mycelium as a conduit to carry information between tree roots.

The aim of this discussion is not to suggest we are all communicating our stressed states like trees but rather to show that these communication systems are amenable to investigation and that it may be possible to understand the impacts of low doses of radiation and other stressors so that predictors of adverse outcomes can be identified. Instead of denying (or passively accepting) uncertainty surrounding the dose effect relationship, we start from the response and work back to model or identify the multiple choices made by the system in arriving at the end result. This is almost like constructing a decision tree in reverse where there are multiple potential finishing states involving multiple decisions which were made, but only one initiating event (the dose deposition in the target). Instead of placing the emphasis on initial conditions as in chaos theory, this approach would require final conditions or outcome to be critical. Rather than fore-casting using dose we need after-casting using response.

## 4. Downstream Events—The Role of RIBE and RIGI

**Figure 2:**
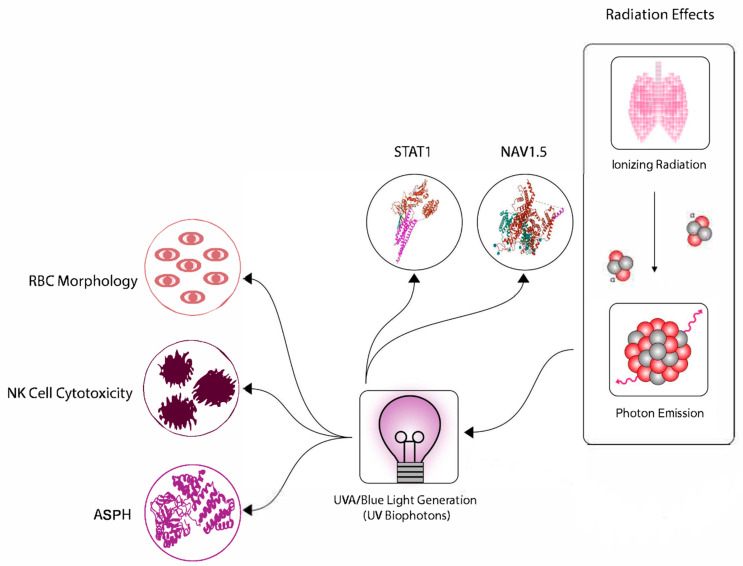
Pathways and processes common to both radiation induced bystander effects, and radiation related chronic fatigue.

In the case of low dose radiation exposures, cancer, cardiovascular disease, foetal abnormality, and chronic fatigue are examples of gross adverse effects [89]. These are thought to result from genomic instability, mutation in critical genes, microenvironment alterations leading to niche compromise, haematological insufficiency and immune dysfunction [7,29,90,91,92,93,94,95,96]. These in turn are attributed to DNA damage, elevated ROS, mitochondrial insufficiency, membrane channel imbalances and failure of “checks and balances” on the rate of specific enzyme reactions, leading to tipping points where metabolic malfunction occurs. In particular, energy production and utilisation by cells is altered [16,97,98]. The energy deposition from low dose radiation and the consequent electromagnetic emissions in the form of light and acoustic signals, can thus be linked with gross outcomes in a way where system level modelling may be applied. Figure 2 presents a possible though at present hypothetical model system linking low dose ionizing radiation exposure and consequent UVA emissions to cancer associated fatigue, radiation associated fatigue and to chronic fatigue syndrome through a variety of stress response pathways involving expression of biochemical markers which are well documented. These include STAT1, NaV1.5 a sodium channel protein located in the cell membrane, ASPH, microRNAs contained in exosomes released by irradiated cells. For a comprehensive review of the role of these agents in fatigue including chronic fatigue syndrome see [99]. Documented downstream events include generalised mitochondrial insufficiency resulting in compromised ATP generation [99]. Specific events then relate to abnormalities in the generation and function of haematopoietic components such as NK cell cytotoxicity and abnormal red blood cell morphology leading to reduced tissue oxygenation, reduced available energy from ATP [99] and consequent conditions favouring Warburg biochemistry with obvious consequences leading to fatigue, and creating a favourable microenvironment where precancerous cells can establish a favourable niche [100,101,102,103].

### Beneficial Outcomes and Adaptive Responses

While radiation protection is concerned with understanding and preventing or reducing the adverse outcomes of radiation exposure, it is well known that beneficial effects can result from low dose exposure. These include adaptive responses which occur when a small “conditioning” dose is given some hours before a challenge dose [104,105]. A related observation is that populations from contaminated sites exposed chronically to low doses of radiation can exhibit relative resistance to an acute high dose exposure [106,107]. This has also been shown to occur in bystander individuals not from the chronically exposed population if they were in communication with exposed individuals [108,109,110]. These observations raise important questions for how we do risk assessments particularly in the environment [111] but also in human medicine discussed later. Related to adaptive response is the phenomenon of “induced radioresistance” (IRR). This is usually coupled with low dose hyper-radiosensitivity (HRS). The effect manifests as a breakpoint in a conventional survival curve where in the low dose region a steep decline in survival occurs with increasing dose. As the dose increases past the breakpoint, the rate of change of survival with dose falls, leading to the conclusion that some dose threshold has been passed where a new repair or recovery mechanism is activated [112,113]. Again the discontinuity posed issues for low dose risk assessment as linearity cannot be assumed. Possibly the most important low dose beneficial effect is hormesis. This has been worked on extensively and the field is the subject of multiple reviews by Calabrese (e.g., [114,115]) who makes the case that the fundamental basis of radiation protection which ignores beneficial effects, is flawed and built on a false analysis of the data available [116]. The rights or wrongs of this debate are not relevant to this paper but the fact that multiple outcomes are possible and very well documented after low doses is critical to our proposed model.

## 5. Proposal of Common Mechanism

The above discussion suggests common mechanisms may underlie various low dose radiation induced phenomena although it is important to note that the range of doses and dose rates over which these effects manifest are not fixed and therefore the low dose effects mentioned may not be mutually exclusive. The outcomes also may also vary. These include adverse effects such as induction of CFS and carcinogenesis but also adaptive responses such as darkening of the skin due to melanin induction, which absorbs UV, adaptive induction of anti-oxidant pathways [117,118,119] and elimination or rehabilitation of potentially dangerous cells by apoptosis or autophagy before they can establish in a favorable microenvironment [120]. However, identifying multiple outcomes does not help to predict risk unless robust markers or models can be developed. The pathways discussed lead to specific outcomes in biochemical terms but it is important to know how these relate to gross outcomes such as cancer or CFS when there are multiple points between detection of a marker or pathway in a cell and the expression of systemic disease in a whole organism.

The next question is how to integrate low dose and NTE radiobiology into practical concerns about radiation risk from low dose exposure?

## 6. Relevance and Impacts in Radiation Protection and Medicine

Currently data to inform radiation protection risk comes from two main sources

1. Accidental or deliberate exposures of humans. Examples include the Hiroshima and Nagasaki bombings, the Chernobyl and Fukushima nuclear accidents the Techa river communities, atomic veterans, medical exposures and a new initiative—the Million Person Study [121,122,123,124].
2. Experimental data from animal studies such as the 7 million mouse study, the Beagle inhalation study or the EULEP database now known as STORE [125,126,127].

ICRP currently rely almost exclusively on the life span study (LSS) of Japanese A-bomb survivors as a “gold standard” which in the higher dose ranges support the linear-no-threshold (LNT} model of cancer risk from radiation exposure [128]. The issue accepted by everyone, is that while the data for people exposed to doses greater than about 50–100 mGy do support a linear relationship between dose and cancer risk, the data for lower doses in the range experienced during medical diagnostic imaging, are highly uncertain and almost any line could be theorised to fit. It is hoped that data from the Million Person study, which is examining health data from multiple cohorts of North Americans will answer the key question of the risk associated with prolonged rather than brief exposure. The study also hopes to settle the question of whether LNT is a useful model for chronic low dose exposure risk prediction. The study involves five categories of workers and veterans exposed to radiation from 1939 to the present [121].

The history of how LNT moved from being a “theory” to a “hypothesis” and finally a “model” is interesting and mirrors the developments in our understanding of low dose radiobiology and the roles of genetics, epigenetics and environmental factors in determining outcomes. Even this history is controversial as the three selected review papers [129,130,131] demonstrate!

The model consists of a line extrapolated through zero relating dose and cancer incidence attributable to the dose. At zero dose there are zero cancers attributable to radiation. LNT was historically developed based on two factors: First is the A-bomb data as discussed earlier which suggested a linear relationship if the available data were extrapolated back to low doses. The second factor was theoretical—at the time the dose effect relationship was emerging from epidemiological data, studies in radiobiology and radiation carcinogenesis had determined a central role for DNA as the target and DNA double strand breaks (DSB) as the key lesion responsible for producing cancers from radiation [89]. This made the LNT theory as it was then called, a neat explanation combining logical assumptions and experimental data. If there is no strand break there is no risk but even a single DSB carries the tiniest possibility that a cancer predisposing mutation could occur.

**Figure 3:**
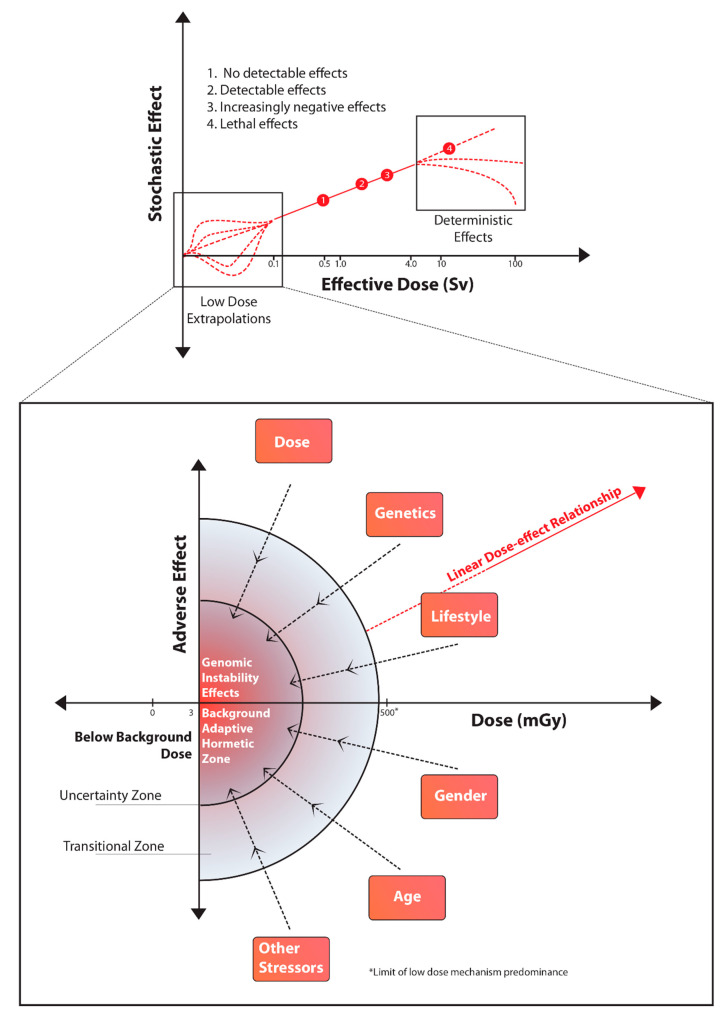
Non-linearity in the low dose region of the dose response curve with factors impacting outcomes.

The complexity of low stressor dose response was not appreciated at the time when LNT was developed as the central tool in radiation protection. Epigenetic mechanisms including signaling were not accepted as critical determinants of outcomes. Now, the type of relationship shown in Figure 3 is thought to be more meaningful. This figure taken from [132] makes the important point that LNT is “good for purpose” only in a limited dose range. At extremely high doses or extremely low doses it does not work. The low dose range however, includes the range of importance in diagnostic imaging and most environmental discharges [133]. The figure draws attention to the need for establishing boundaries in radiation protection, within which certain relationships hold but outside of which they do not.

The concept of boundaries outside of which dose response relationships do not hold is an important contributor to defining the level of uncertainty. It also helps to fix the doses above and below which relationships such as LNT do hold. The concept helps to resolve the controversies surrounding the use of LNT. It also allows regulators to consider biological plausibility as an important factor—the DNA centric paradigm underlying LNT can now be seen as an explanation for the relationship in a defined range. Below and above this range other biological mechanisms dominate the response. This begs the question of whether we can identify and validate plausible biological mechanisms, which could define the relationship in the low dose range? If so, can these be defined well enough to reduce the uncertainty surrounding low dose outcomes? Can they be validated using available epidemiology?

The answers to these questions are probably yes, no and not yet! Yes we do have data in vivo and in vitro confirming that different mechanisms predominate after low dose exposure. These were discussed earlier and are defined in Table 1. We do know the dose response relationship for each of these effects so can estimate the contribution of each effect to the over all outcome. However, to reduce the uncertainty, it is necessary to be able to say which mechanism will predominate in a particular individual in a defined scenario and this we cannot currently do because we cannot estimate the importance of the interplays between the effects. In precision medicine, this is being attempted in certain situations where omics technologies and lifestyle factors are used to plan treatments but in low dose radiation exposures the stochastic nature of radiation interaction with the genome, means it may be intrinsically impossible to predict whether, for example a hormetic outcome or a low dose hyper radiosensitivity response will result from the exposure. Another layer of complexity is that an adverse outcome at the level of the individual cell could be beneficial at the level of the individual person if it eliminates a damaged cell. In regard to the third question, epidemiology is regarded as a blunt tool following low dose exposures [134]. The problem is that cancer is very common, meaning that to detect an excess risk requires vast numbers of people to be followed in a study. It has been estimated that to detect a statistically significant excess risk for solid cancer after 100 mGy exposure would require over a million people exposed to this dose. This is why the million person study was established. It is one attempt to use epidemiology to assess effects of chronic low dose but may take several years to provide answers and will only give probabilities of getting a disease after chronic exposure.

Given the above analysis, we contend it is impossible using current methods to give definitive answers about individual risks—probably because there are no absolute answers. One approach might be to try to calculate an uncertainty potential or add an uncertainty factor to risk of harm equations. Trying to achieve this means grappling with a major cause of the uncertainty which is that in the low dose region there are competing stressors many of which as discussed earlier, operate through common mechanisms including oxidative stress or energy depletion. How do we tease out whether a patient’s CFS or cancer was caused by, for example, a chemical in the environment or the radiation in the environment? We can say it is plausible that radiation may have caused the condition but we cannot attribute causation to radiation in this situation. How therefore can we deal with uncertainty caused by the multitude of stressors that can induce effects similar to low dose effects?

Radiation protection data are currently insufficient therefore, for chronic exposures, for very low doses, low dose rates, and for multiple genetic backgrounds and in environmental radiation protection for species, communities and ecosystems. The laboratory animal experiments have looked at various dose regimes but it is argued that mouse data or beagle data cannot be assumed to hold for humans or other species particularly in the natural environment. For much of the experimental data generation, inbred murine strains were used and they do not adequately inform about individual radiosensitivity in outbred humans. In the environment, the amalgamation of multiple datasets for multiple species allows the generation of species sensitivity distributions (SSD) which could be a promising way to allow impacts to an ecosystem to be evaluated [135,136].

Major assertions advanced by groups challenging the LNT model in both directions include: either the radiation is more dangerous in the low dose region and due to the HRS phenomenon and genomic instability occurring after low dose exposures, or there is a threshold below which LNT does not hold and that all the efforts to keep radiation doses lower are a waste of money or positively harmful. It is important to accept that no one has established a “carcinogenic dose threshold”. All we can say is that causation cannot be definitively attributed to radiation below 100 mGy, due to the uncertainty in the response below this dose. Additionally, this is precisely the point!

Where human health is not an issue or where individual survival in populations of non-humans is not an endpoint of concern, it is easier to use thresholds defined as “acceptable levels of harm” such as PNEDR meaning “predicted no observable effect dose/dose rate”. For both human and non-human studies, datasets which might be appropriate for looking at low dose risk include those from legacy sites such as Chernobyl, Maralinga, Fukushima or Techa River, or those from medical exposures such as CT scans, radiological exams and radiotherapy, including large human mega studies in Europe. The data from Chernobyl revealed an excess risk of thyroid cancer in young people [137]. This has been linked to iodine deficiency in the soils which meant that the thyroid took up radioactive iodine when it became available [138]. It has also been linked to consumption of wild fruits and mushrooms [139]. The excess of thyroid cancer is the only officially recognised excess cancer outcome of the Chernobyl disaster, although in Belarus and Ukraine, there is some evidence for excess breast cancer and cardiovascular disease [140,141,142]. The problem is establishing causation in a population of mainly impoverished people with unhealthy lifestyles. Similar problems exist with the nuclear tests in Australia at Maralinga where indigenous peoples were exposed. No excess cancers have been linked to the Fukushima accident—here the triple stresses of the earthquake, tsunami and radiological accident, combined with the disruptive effect of the mass evacuations, loss of livelihoods and habitat destruction during rehabilitation of contaminated lands, makes it impossible to assign causation in any meaningful way. The Techa River cohort received significant doses over long periods related to release of radioactivity into the river Techa from the plutonium production sites associated with the Soviet nuclear testing programme. This population has been studied in great detail and again at high doses the data do support the LNT model [143]. However, the data have very large uncertainties due to the difficulty of estimating dose retrospectively, migration of people thus lost to follow-up, unhealthy lifestyles, and multiply contaminated sites. Many studies of contaminated sites have been done looking at animal and plant populations. Some of these do show effects such as variation in wing patterns of butterflies or tumours in Chernobyl birds [144,145,146] but relating these to harm in humans is probably not useful. The data however have been used in efforts to develop tools for environmental risk assessments [147].

Efforts to assess low dose and chronic exposure risks have also been made by looking at nuclear workers. For example, the15 country study which pooled data from nuclear workers from 15 countries to give a total of over 400,000 workers [148]. This is highly controversial although it supported an LNT model in the low dose region looking at leukaemia incidence in the workers. The cause of the controversy is because if the Canadian cohort was removed, there was no excess risk below 100 mSv. Various suggestions to explain the discrepancy include, smoking and also the nature of dose record keeping [149]. Other intensely studied groups include the atomic veterans. These were military personnel sent to witness the nuclear tests. They were deliberately exposed in the US during exercises to determine whether soldiers could fight successfully after a nuclear explosion [150]. Similar exposures were suffered by British Commonwealth veterans and French veterans however no (accurate) dosimetry was done. The relevant governments either compensated all who got any of a list of named diseases without determining causation, or as in the case of the UK government, denied any possibility that any of the diseases suffered by the veterans, were linked to exposure to radiation during the tests [151].

Another increasingly important source of low dose exposure to humans is during medical diagnostic imaging. Several studies have followed people who had CT scans or other imaging procedures. For recent reviews see [135,152]. These studies suggest cancer risks increase with increasing doses over about 100 mGy with increasing evidence for risks below 100 mGy. The results of studies which looked at the outcomes following paediatric CT scans are variable and contradictory [153,154]. All this just adds to the evidence that low doses produce unpredictable outcomes when viewed at the level of the individual. Probabilities of various outcomes can be more easily developed for populations as long as robust markers of response are available.

## 7. A Way Forward? The Variable Response Model

**Figure 4:**
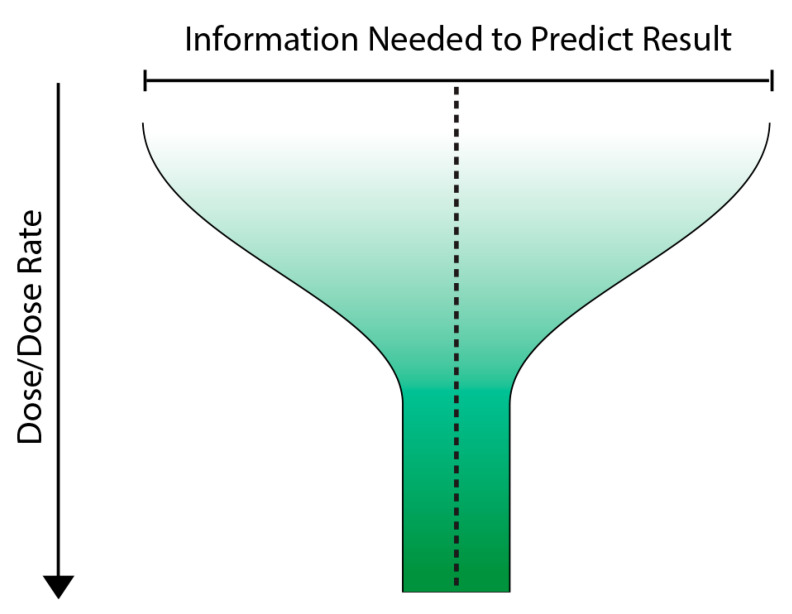
A simple cartoon depicting the Variable response model (VRM) where as the dose increases, the amount of information needed to predict outcome decreases.

A possible way forward is to use multidimensional modelling to build up a more holistic picture of the key determinants of response. This could include time, dose, system level and outcome as initial parameters and build on this to include other determinants of outcome as they were identified. Such an approach is used by Carreon-Ortiz, & Valdez cited above [86], A key feature of the model which we have called the variable response model (VRM) would be its emphasis on outcome or response not as a surrogate for dose but as the starting point of interest. We propose the use of the term infosome rather than infochemicals to highlight the fact that radiation-induced communication signals include physical as well as chemical agents. The VRM could be used to link responses associated with disease to a possible role of low dose ionising radiation exposure in populations. We are not talking about replacing dose but rather suggesting that dose alone is an inadequate predictor of response below 100 mGy. Considering the infosome might make the response more predictable. Figure 4 presents a very simple cartoon of the VRM where as dose increases, the amount of information needed to predict the outcome decreases and ultimately at high doses, dose is the main predictor of outcome. We see this as a funnel which narrows or streamlines the options as the dose increases.

A similar approach is to use system biology modelling which gets away from estimating outcomes on single cells or individuals and instead develops multi-level models which input various parameters known to be relevant. The adverse outcome pathway analysis (AOP) is now being used in human and environmental toxicology. This assesses multiple levels from genes to gross pathology of organs to identify molecular initiating events and relate them to pathological outcomes. Would these approaches be of any value in reducing uncertainty? It is possible they would help in environmental analysis but it is questionable whether the approach would help resolve uncertainties in estimating medical imaging associated risks or whether they would be useful in diseases where the aetiology in unclear such as CFS. In these cases the VRM is likely to be more useful given its holistic framework and approach.

## 8. Conclusions

The main conclusion of this paper is that multiple outcomes are possible after low dose exposure to stressors. These include stimulation of processes that are generally beneficial such as immune stimulation. However, stimulation of processes that could lead to adverse outcomes such as mutation tolerance, leading to genomic instability, can also occur. The challenge is to move from a thought framework developed for high dose exposures, where dose is the key predictor of outcome to a response-based framework which takes the outcome as its starting point.

## Figures and Tables

**Table 1:** Definitions of Low Dose Mechanisms.

Direct Effects
Adaptive response: A low “priming” dose of radiation induces protection against a later higher dose.
HRS/IRR: Hyper-radiosensitivity after low dose exposure is lost as the dose increases and a region of induced radioresistence is seen at higher doses
Hormesis: A beneficial effect of low dose exposure is seen compared to unirradiated controls.
**Non-Targeted Effects**
Bystander effect: An effect detected in non-exposed cells which received signals from irradiated cells. Can also apply to tissues and organisms.
Genomic instability: Detection of chromosomal or other DNA damage in progeny of irradiated cells which was not present in the first post-irradiation mitosis.
Lethal Mutations: A form of genomic instability leading to a permanently reduced plating efficiency in progeny cell lineages which survived irradiation.

## Data Availability

Not applicable.
